# Mobile health interventions on vaccination coverage among children under 5 years of age in Low and Middle-Income countries; a scoping review

**DOI:** 10.3389/fpubh.2025.1392709

**Published:** 2025-01-28

**Authors:** Olanrewaju Onigbogi, Omobola Yetunde Ojo, Ulla-Mari Kinnunen, Kaija Saranto

**Affiliations:** ^1^Department of Community Health and Primary Care, University of Lagos, Lagos, Nigeria; ^2^Department of Health and Social Management, University of Eastern Finland, Kuopio, Finland; ^3^Department of Family Medicine, Indiana University, Indianapolis, IN, United States; ^4^Department of Community Medicine and Primary Care, Federal Medical Center, Abeokuta, Nigeria

**Keywords:** Low- and Middle-Income countries, mHealth, scoping review, vaccination, under five, children

## Abstract

**Objectives:**

Increased mobile phone use in Low- and Middle-Income countries (LMIC) has led to suggestions that health interventions using mobile phones can help solve some health problems. Vaccination has been shown to be an effective means of improving health outcomes. However, vaccination coverage in many LMIC has been generally low. The aim of this study was to synthesize evidence concerning the context, mechanisms, and outcome elements of mobile health interventions in improving vaccination coverage among children under 5 years of age in LMIC.

**Methods:**

A search conducted using PubMed, Web of Science, ScienceDirect, CINAHL, Embase, and the Cochrane library led to 27 studies included in the final analysis out of 357 identified articles.

**Results:**

Twenty-one studies were from Africa, four from Asia and two studies were from Latin America and the Caribbean. Short Message Service (SMS) intervention was used exclusively in 21 studies while six studies used a combination of SMS and phone calls, and one intervention was based only on phone calls.

**Conclusion:**

The results from most studies suggest an improved uptake of vaccination with mobile health interventions. However, there is a need for further research to quantify the impact of these interventions and determine the most effective strategies.

## 1 Introduction

Mobile health (mHealth) technologies have been implemented in Low- and Middle-income countries (LMIC) to address public health challenges (1, 2). Many of these technologies were designed to either influence patients’, caregivers’, or health workers’ behavior or impact health outcomes (3–5). The World Health Organization (WHO) Global Observatory for eHealth defines mHealth as a medical and public health practice supported by mobile devices, such as mobile phones, patient monitoring devices, personal digital assistants, and other wireless devices (6).

According to the International Telecommunication Union, the number of mobile users worldwide in year 2021 stood at 7.1 billion and is projected to reach 7.49 billion by 2025 (7, 8). The majority of smartphone users are expected to be in developing countries (9, 10). In addition, mobile phone subscriptions in many LMIC have been shown to expand faster than other infrastructure (11, 12). Moreover, mobile phone access in these countries is greater than 60% because of personal or shared ownership (13). Characteristics such as mobility, instantaneous communication, relative cheap cost, and ability to use for long periods without electric power and in comparison, to other communication infrastructures may be contributing to this expansion (13).

mHealth activities include the use of applications and technologies such as voice, text messaging, also referred to as Short Message Service (SMS), and multimedia message services (14). Therefore, the relative ease of use of mobile phones for SMS has the potential of transforming maternal and child health services, especially in LMIC where investment in health care infrastructure is generally low. mHealth interventions could impact the attainment of the global targets 3.1 and 3.2 of reducing maternal, neonatal and under 5-year mortality under the Sustainable Development Goals 3 (15).

In addition, vaccines are generally believed to set one of the highest standards on ‘return on investment’ in the field of public health because they are highly cost-saving. Vaccination has been proven to be a cost-effective means of improving health outcomes in many parts of the world. A study in the United States (U.S) estimated that every dollar spent on childhood vaccination resulted in a US$3 savings from a payer perspective and a US$10 savings from a societal perspective (16). External factors such as social and political disruptions, disruption of household integrity, school absenteeism, health care utilization and long-term/on-going disability are usually considered in assessing the success of vaccination programs (17–21). Childhood vaccination with the measles antigen is believed to have long-term benefits of preventing all-cause infectious disease by preventing measles-associated immune memory loss and protecting polymicrobial herd immunity (22). Extra attention has been paid to the measles virus in comparison to other viruses responsible for childhood illnesses because of its capacity to cause long-term damage to the immune system, leaving people vulnerable to other infections (22).

Despite the obvious benefits of childhood vaccination, morbidity and mortality from vaccine-preventable diseases continue to challenge the health care systems in LMIC due to limited health infrastructure and a dearth of qualified personnel (23–25). There is an urgent need to address worldwide disparities in health outcomes by exploring innovative means of improving vaccination coverage (26, 27).

The recent increase in reports on the use of mHealth applications to facilitate vaccine uptake has resulted in questions about how effective these approaches are in LMIC (28, 29). Reminders via SMS have been used in some interventions to address memory lapses which is a common reason given for the failure of mothers or care-givers to present their children or wards for vaccination during the next due date (30, 31). mHealth techniques have also been used to exchange health information with caregivers about vaccine dosages and adverse drug reactions. The general belief is that the use of mHealth interventions has the potential to address some of the issues and challenges related to failure of caregivers to take their wards for vaccination (32, 33). However, there are other factors that researchers had observed limit vaccination access in these settings: these include the level of literacy, role of family decision makers, poverty, lack of access roads, and concerns about the safety of the women and children who ought to be vaccinated (33). The possibility of using mHealth interventions to circumvent some of these barriers to vaccination, could lead to improvement in coverage especially in LMIC. Countries that fall into LMIC classification by the World Bank have an average per capita government spending on health of about US$15 in 2018 and received donor financing for vaccines through the Global Alliance for Vaccine (34). In addition, there have been reports of socioeconomic inequalities in child vaccination in these countries (35, 36). Previous reviews, show that systematic reviews and meta-analyses demonstrate trends and design models capable of improving the impact of mHealth interventions on health care outcomes including vaccination (37, 38). These reviews tend to look at the effectiveness of interventions (quantitative) or experiences (qualitative). Furthermore, some vaccination interventions with mixed information delivery modes have been shown to have an enhanced effect on coverage (39, 40). Some other studies have shown an increase in community participation in vaccination among mothers and caregivers by virtue of combining other communication methods with the vaccination process especially if it involves development and pilot testing phases (41–43). However, many of these studies have failed to examine the context under which these programs were undertaken. Some of these studies also failed to define the mechanism adopted for the mHealth intervention, hence the need for a scoping study addressing these gaps identified in literature as its results may be of benefit in improving coverage for new emerging diseases such as COVID-19 and other coronaviruses especially in LMIC (44, 45).

This scoping review therefore had the aim of synthesizing evidence on a general level concerning the context, mechanisms, and outcome elements of mHealth interventions in improving vaccination coverage among children under 5 years of age in LMIC.

## 2 Methods

### 2.1 Study design

This study used a scoping review methodology. Using the framework that had been adopted in similar studies, we did the following: (1) identified the research question; (2) identified relevant studies; (3) selected studies; (4) charted the data; (5) collated, summarized, and reported the results (46–50). For the purpose of this review, LMICs were defined in terms of the World Bank classification of countries on the basis of the Gross Domestic product (51). The review is driven by the primary question: What mHealth interventions have been used to improve vaccination coverage in children 0–5 years in LMIC?

### 2.2 Participants’ inclusion and exclusion criteria

The inclusion criteria are identified in relation to the research question with the help of PCC (Population, Concept, and Context) (50). The population of interest is children aged 0–5 years, the concept is the impact of mHealth on vaccination coverage among children 0–5 years, and the context is existence of conventional vaccination schedules in LMIC (Table 1).

**Table 1:** Inclusion and exclusion criteria.

	Inclusion criteria	Exclusion criteria
Population	Children aged 0–5 years	Children older than 5 years
Concept	mHealth interventions for vaccinations	Other interventions not using mHealth for vaccinations
Context	Low- and Middle-Income Countries as ranked by World Bank	Countries ranked as High-Income Countries by the World Bank
Language	English	Language other than English
Years of database search	Studies published between 1^st^ January 2000 -30^th^ October 2024	Studies published before 1^st^ January 2000

### 2.3 Search strategy

Medical Subject Headings (MeSH) and non-MeSH terms were used to search selected databases. MeSH tterms used in the search included mHealth, telehealth, mobile Health, eHealth, mobile phone, cellular phone, “cell phone,” “text message,” Africa, Asia, South-East Asia, Sub-Saharan Africa, Far East and Middle East, Latin America, Hispanic and Caribbean Islands, Low- and Middle-Income Countries, LMICs, vaccination, immunization, and inoculation. Pertinent terms were selected after two separate internal discussions and then strung together with Boolean operators ([AND], [OR]). The screening for applicable titles and abstracts was guided by the Joanna Briggs Institute Guidelines (52). Application of inclusion criteria ensured that the content of the included studies was relevant to the aim of the study and the research question. The articles were then assessed for relevance.

### 2.4 Data sources

The following databases were used in the search: PubMed, Web of Science, ScienceDirect, CINAHL, Embase and Cochrane library. Other sources of information used were University of Eastern Finland library electronic thesis and gray literature online resources. The search was from 1^st^ January 2000 to 31^st^ October 2024. The review was limited to publications in English language only. Details of the search strategy used for each database is outlined in Supplementary Table S1.

### 2.5 Data extraction

**Figure 1:**
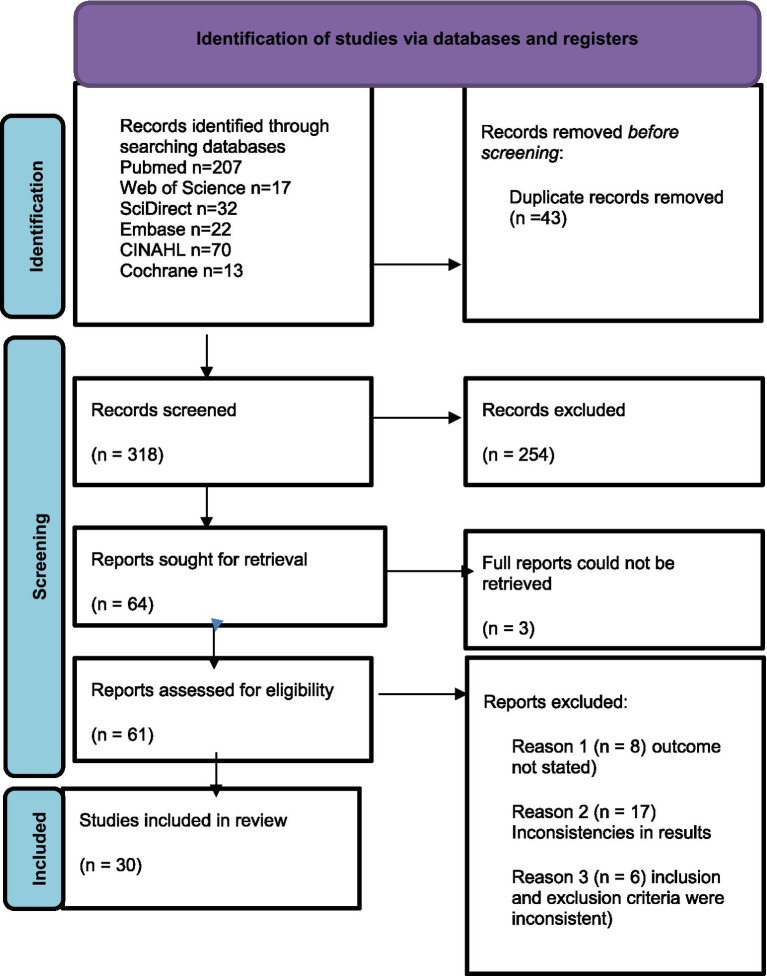
PRISMA flow diagram.

Two researchers independently conducted the initial screening of titles and abstracts of articles identified through the search. Reference lists from included studies were used to identify 36 relevant studies which were added to the search. A data extraction was done manually using the following information: the author’s name, year of study, country where the study was conducted, the study design, study population, and outcome of interest. The primary and secondary reviewers used the inclusion criteria to determine eligibility of the studies and subsequently conducted full-text screening of all eligible articles. Articles were selected on a minimum agreement of at least 50% between the two reviewers. The researchers then had agreement meetings to decide on what studies to keep. The reporting of this study process followed the recommendations of the Preferred Reporting Items for Systematic Reviews and Meta-Analyses Extension for Scoping Reviews (PRISMA-ScR) (47) (Figure 1). A narrative report was produced to summarize the extracted data. These results were described in relation to the research question and in the context of the overall study purpose.

## 3 Results

Thirty-one studies were included in the final analysis out of a total of 361 articles initially identified. The most documented mHealth applications in use were one-way text-message and phone reminders to encourage vaccination follow-up appointments (Table 2). There were eight studies from Nigeria, four from Kenya, three from Ethiopia, two from Guatemala, two from Pakistan, while Zimbabwe, Cote D’Ivoire, Tanzania, Philippines, China, India, Burkina Faso and South Africa had one study each reviewed (53–79).

**Table 2:** Summary of reviewed studies (table order according to intervention method used).

Ref. No	Authors, year of publication, country	Study design	Study population (*n* = sample size)	mHealth intervention used	Key findings
(53)	Bangure et al. 2015, Zimbabwe	Randomized Controlled trial (RCT)	Mothers and caregivers (*n* = 304)	SMS text reminders	Immunization coverage and adherence to schedule was better in the intervention group when SMS reminders were sent
(54)	Kazi et al. 2014, Pakistan	Descriptive study using random sampling method to recruit participants	Caregivers of young children (*n* = 28)	SMS-based monitoring	Poliomyelitis vaccine coverage using the SMS system were similar to those estimated by interviewing those caregivers who never responded to the SMS messages.
(55)	Wakadha et al. 2013, Kenya	Intervention study	Mothers (*n* = 72)	SMS reminders	Addition of cash incentives was found to be an effective strategy to increase immunization coverage
(56)	Domek et al. 2019, Guatemala	RCT	Mothers and care-givers (*n* = 1,080)	Mobile calls and SMS	In rural areas, landlines were used. Mobile ownership was higher in urban areas with these participants receiving reminders and having higher vaccination rates compared to participants from the rural areas.
(57)	Nguyen et al. 2017, Vietnam	Pre- and post-intervention study	Mothers (sample size not stated)	SMS	Marked increase in full immunization coverage with participants willing to pay for SMS reminders
(58)	Seth et al. 2018, India	RCT	Mothers (*n* = 608)	SMS	Automated mobile phone reminders increase infant immunization coverage among Indians
(59)	Kazi et al. 2018, Pakistan	RCT	Mothers (*n* = 356)	SMS	No significant difference observed for EPI 10 and 14- week vaccination schedule for the children
(60)	Coleman et al. 2020, South Africa	Intervention study	Pregnant women (*n* = 87)	SMS	Health information text messages sent to women during pregnancy led to positive adherence to all first-year vaccinations for infants
(61)	Oladepo et al. 2021, Nigeria	RCT	Mothers (*n* = 3,440)	SMS	Messages increased awareness of immunization dates, assisted in timely completion
(62)	Mekonnen et al. 2021, Ethiopia	Cross-sectional study	Mothers (*n* = 456)	SMS	Majority of mothers indicated the intention to use text message reminders for child vaccination
(63)	Ekhaguere et al. 2019, Nigeria	RCT	Households (*n* = 600)	SMS	Paired automated call and text reminders significantly improved immunization completion and timeliness
(64)	Dissieka et al. 2019, Côte d’Ivoire	RCT	Mothers (*n* = 798)	Phone calls and SMS	Providing mothers with SMS reminder messages increased the proportion of child immunization
(65)	Brown et al. 2016, Nigeria	RCT	Mothers and care-givers (*n* = 214)	Phone calls	Increased coverage rates relative to the usual care when receiving phone call reminders 2 days and 1 day before a vaccination appointment (Relative risk 1.72, 95% CI 1.50–1.98).
(66)	Garcia-Dia et al. 2017, Philippines	Descriptive study	Parents (*n* = 59)	Call reminders and SMS	Parents who received call reminders alone, brought their children for measles, mumps, and rubella immunization on a timelier basis
(67)	Yunusa et al. 2022, Nigeria	Quasi-experimental study	Care-givers (*n* = 541)	Call reminders and SMS	Mobile phone reminders were effective and improved the rate of completeness of the pentavalent vaccine in Kano, Nigeria
(68)	Chen et al. 2016, China	Cluster randomized controlled trial	Mothers (*n* = 2,611)	SMS	Smartphone application improved immunization of children in rural Sichuan Province, China.
(69)	Atnafu et al. 2017, Ethiopia	RCT	Mothers (*n* = 3,240)	SMS	mHealth intervention improved childhood vaccination and overall health service delivery
(70)	Shiferaw, S. et al. 2016, Ethiopia	Prospective Controlled Evaluation	Mothers (*n* = 1970)	SMS	mHealth Intervention improved Postnatal Care Utilization including vaccination coverage increased in health centers
(71)	Kawakatsu et al.2020, Nigeria	RCT	Caregivers (sample size not stated)	SMS	SMS appointment reminders increased vaccination uptake in Lagos
(72)	Schlumberger et al. 2015, Burkina Faso	RCT	Mothers of newborn babies (*n* = 523)	SMS	There was positive impact on the Expanded Program on Immunization when SMS were sent via Computerized Immunization Register
(73)	Haji et al. 2014, Kenya	RCT	Parents (*n* = 1,116)	SMS	Children whose parents received text messages were less likely to drop out compared to controls (OR 0.2, CI 0.04–0.8). There was no statistical difference between those who received stickers and controls (OR 0.9, CI 0.5–1.6)
(74)	Gibson et al. 2017, Kenya	RCT	Mothers (*n* = 2018)	SMS	There were four groups: control, SMS only, SMS plus a 75 Kenya Shilling (KES) incentive, and SMS plus 200 KES (85 KES = USD$1. Mobile phone-delivered reminders and incentives improved childhood immunization coverage and timeliness in intervention group
(75)	Eze et al. 2015, Nigeria	RCT	Caregivers (*n* = 905)	SMS	Enhanced routine immunization performance using innovative technology in an urban area of Nigeria
(76)	Ibraheem et al. 2021, Nigeria	Quasi-experimental study	Caregivers and child pairings (*n* = 560)	Calls and SMS	Four (three interventions, one control) groups, each consisting of 140 participants. Improved childhood routine vaccination timing and completion in Ilorin, Nigeria.
(77)	Kagucia EW et al. 2021, Kenya	RCT	Mothers (*n* = 537)	SMS	179 infants were enrolled into each of the three study arms Mobile phone delivered reminders and unconditional incentives increased measles-containing vaccine timeliness and coverage.
(78)	Osterman J. et al. 2019, Tanzania	Quasi-randomized controlled trial	Mothers (*n* = 400)	SMS	Timeliness of vaccinations among their children at 6, 10, and 14 weeks.
(79)	Uddin et al. 2016, Bangladesh	Quasi-experimental study	Mothers (sample size not stated)	SMS	Mobile phone intervention improves vaccination coverage in rural hard-to-reach and urban street dweller communities in Bangladesh.
(28)	Gilano et al. 2024 African countries	Systematic review and meta-analysis	Mothers	SMS	A systematic review which showed that the application of mHealth could potentially improve childhood vaccination in Africa. Study found to have increased childhood vaccination among children whose mothers were motivated by mHealth services.
(80)	Louw et al. 2024 (Upper, middle, and low-income countries)	Systematic review and meta-analysis	Mothers	Mobile phone text message reminders	Mobile phone text message reminders have a small but positive effect on vaccination uptake.
(39)	Eze et al. 2021. Eleven Low and Middle-income countries	Systematic review and meta-analysis	Mothers (13 RCTs and 5 non-RCTs)	SMS	SMS reminders can contribute to achieving high and timely childhood immunisation coverage.

The most commonly used mHealth intervention was SMS without a phone monitoring component which was used to determine the vaccination rate or improve coverage in 19 studies (54, 57–63, 68–74, 76–79). Four studies used a combination of SMS and phone calls to achieve similar objectives among participants (56, 64, 67, 75). In one study, the researchers interviewed caregivers by sending SMS in order to determine the poliomyelitis vaccine coverage by vaccinators during Supplemental Immunization Activities (53). A Nigerian study used only mobile telephone calls to determine the coverage of vaccination among participants (65). Most participants were a combination of mothers and care-givers as observed in 24 studies. Only two studies were designed with both parents as participants (66, 73) and one study focused on households (63). Moreover, results of the effect of mHealth on vaccination outcomes from studies conducted in countries classified by the World Bank as lower middle-income countries (Nigeria, Tanzania, Kenya, Côte d’Ivoire, Philippines, Pakistan) were identical to those obtained from South Africa, Guatemala, China, Vietnam, India which are classified as upper-middle income countries. Furthermore, countries classified as low-income countries such as Zimbabwe, Burkina-Faso and Ethiopia did not have markedly different results despite the variety of study methodology adopted by the researchers.

There were 18 randomized controlled trials (RCT), three quasi-experimental, and three intervention studies. In addition, there were two cross-sectional/descriptive studies and one prospective controlled evaluation study. The total participants in the studies ranged 28 (54) to 3,440 (61). Four studies each were conducted in years 2017 and 2019 while Year 2015, 2016, 2018, and 2021 each contributed three studies.

Most of the RCT studies reported the use of phone calls or SMS reminders to increase vaccination coverage (53–63). Three of these studies (two in Kenya and one in Nigeria) included monetary incentives to increase coverage among participants (58, 65, 72). Twenty-one of the studies were conducted on SMS vaccine reminders in Africa, out of which 18 revealed either an increase in vaccination coverage, decrease in dropout rates, increase in completion rate, or a decrease in delayed vaccination (53–60, 62–79). Six studies focused on SMS reminder systems as an intervention, with no reminder system as the control. However, four studies added other forms of intervention alongside SMS reminders (59–65). A Zimbabwean RCT showed that immunization coverage and adherence to immunization schedule was higher among those in the SMS intervention group in comparison with the control group. SMS reminders were sent to parents (n = 152) when their baby was 6, 10, and 14 weeks old, in addition to routine health education. The control group received health education alone (n = 152). At all three time points, the percentage of children fully vaccinated with the relevant dose of polio, pentavalent, and pneumococcal vaccines was significantly higher in the intervention group compared to the control group (*p* < 0.001), and the delay in receiving the vaccinations was significantly less in the intervention group compared to the control group (*p* < 0.001) (59). Another RCT conducted in Nigeria identified increased coverage rates relative to usual care when receiving phone call reminders 2 days and 1 day before a vaccination appointment (relative risk 1.72, 95% CI 1.50–1.98) (67). A similar RCT by Osterman et al. in Tanzania found that SMS reminders increased the odds of vaccination uptake in both urban and rural areas; odds ratio (OR) 2.3 (95% CI 1.1–5.5) and OR 3.6 (95% CI 1.5–8.9), respectively (78). Chen et al. (2016) in another RCT recruited village doctors to assess the effectiveness of a smartphone application on improving vaccination coverage in China with the primary outcome as full vaccination coverage and the secondary outcome as coverage. The study found that smartphone applications improved immunization of the children (68).

Another RCT conducted in year 2017 among 3,242 Ethiopians indicated that SMS interventions resulted in an increase in utilization of maternal health care services with no significant impact on childhood immunization (70). An RCT using 3,440 Nigerian women revealed that messages increased awareness of immunization dates and assisted in timely completion of vaccination (71). In addition, Ekhaguere et al. in another RCT conducted in Nigeria discovered that pairing automated call and text reminders significantly improved immunization completion and timeliness (63). An RCT conducted in Côte d’Ivoire concluded that voice or SMS reminders provided to mothers with SMS reminder messages increased the proportion of child immunizations (75), while a similar one discovered that delivery of automated mobile phone reminders increased infant immunization coverage (78). Kazi et al. in a Pakistani RCT concluded that although SMS interventions were generally observed to be successful, there was not a significant difference observed between 10 and 14 week scheduled visits (59). However, Domek et al. in a RCT conducted in Guatemala found that SMS messages were more beneficial to most women in urban areas because of high mobile phone ownership (56).

Although they were fewer in number, non-RCT studies also indicated favorable vaccination uptake results. Non-RCT studies included cross-sectional and quasi-experimental studies. For example, a random sampling with SMS-based monitoring of child immunization to ask the caregivers whether their children received vaccines concluded that SMS was an effective strategy to monitor coverage following mass immunization (53). Moreover, Nguyen et al. (2017) reported in a pre- and post-intervention using SMS that texts could increase measles immunization rates and that respondents were willing to pay for the messages (57). Furthermore, a cross-sectional study of 456 women in Northwest Ethiopia found that the majority of mothers had the intention to use text message reminders for child vaccination (62). Garcia-Dia et al. in a descriptive study using call reminders in the Philippines found out that parents who received call reminders alone brought their children for measles, mumps, and rubella immunization on a timelier basis (66). Nguyen et al. (2017) had a similar observation in study conducted among Vietnamese mothers in which an increase in full immunization coverage was observed with participants willing to pay for SMS reminders (57). Coleman et al. also observed a similar trend in South Africa despite conducting the intervention among pregnant women. The researchers observed that health information text messages sent to the women during pregnancy led to positive adherence to all first-year vaccinations for infants (60).

Quasi-experimental studies also indicated a similar trend in the results. Yunusa et al. in their study conducted in Kano, Nigeria found out that mobile phone reminders were effective and improved the rate of completeness of the pentavalent vaccine (67). A similar observation was made in Ilorin, also in Nigeria where caregivers and mothers were put in three intervention and, one control groups, each consisting of 140 participants and researchers observed an improved childhood routine vaccination timing and completion (76). An identical observation was made by Uddin et al. (2016) where mobile phone intervention was found to have improved vaccination coverage in rural hard-to-reach and urban street dweller communities in Bangladesh (79).

Three studies involved systematic reviews and meta-analysis conducted in multiple countries in Asia, Africa and the Americas (28, 39, 80). All the studies reported positive changes in the vaccinated coverage and output of vaccination programs in low -and middle-income countries. The lowest effect was recorded in the meta-analysis of randomized controlled trials of caregivers of children, adolescents, or adults. The researchers concluded that Mobile phone text message reminders had a small positive effect which had no bearing on the intervention characteristics, country setting, country economic status, and vaccination type (80). The most prominent effect was found in a systematic review of 13 RCTs and 5 non-RCTs in which pooled estimates showed that SMS reminders significantly improved childhood immunization coverage. In addition, subgroup analysis showed that SMS reminders were effective in increasing childhood immunization coverage in lower middle-income and low-income countries than in upper middle-income countries (*p* < 0.001) and sending more than two SMS reminders significantly improved timely receipt of childhood vaccines than one or two SMS reminders (39). The individual studies met the criteria used in the systematic reviews to be included, ensuring the review accurately represented the relevant research landscape. In addition, the reviews assessed the quality of each study highlighting discrepancies in quality ratings. All three systematic review studies highlighted consistency or potential differences in comparing the specific outcomes measured in the individual studies to the primary outcomes analyzed in the reviews. Two of the studies involved meta-analysis and another used a risk-bias tool as the statistical method for the analysis. Finally, there was some homogeneity between the results in the systematic reviews studies with individual reviews showing consistent findings and overall conclusions.

## 4 Discussion

The aim of this review was to synthesize evidence concerning the context, mechanisms, and outcome elements of mHealth interventions in improving vaccination coverage among children under 5 years of age in LMIC. The review indicated a generally growing interest by researchers in how mHealth methods could be used to increase vaccination coverage. This is evidenced by a steady increase in number of reviewed articles from 2015 until 2021. This observed interest may be associated with the overall increase in mobile telephone coverage during this time period as reported in previous studies (9–13).

This review included studies conducted in LMIC which have wide socioeconomic inequalities in child vaccination coverage (34, 35). It was impossible to disaggregate data obtained from participants who belonged to the higher socio-economic classes in comparison to those obtained from the lower socio-economic classes in these countries because these categorizations were not available from the reported studies. Therefore, our review was not able to demonstrate a link between mHealth and vaccination either directly or indirectly. Studies with some disaggregation bearing in mind the socio-economic inequalities in LMIC and how it might have affected the vaccination coverage recorded would be helpful for subsequent scoping reviews.

The sample of reviewed studies had more studies from Africa (21) in comparison with four from Asia and two from Latin America and the Caribbean with no studies from Oceania or Europe. The distribution of the studies in this review was not directly proportional to the numerical distribution of LMIC according to the World Bank with 54 countries in Africa, 34 in Asia, 24 in Latin America and the Caribbean, 11 in Europe, and 8 countries in Oceania (51). This observation may be as due to the increased interest of researchers in working on the subject in African countries because of the general perception of poorer health outcomes on the continent.

Another interesting point for consideration is the possibility for interventions with mixed information delivery modes to have an enhanced effect. This trend has been seen in previous studies (39, 40). In this review, interventions that combined texting and phone calls all reported improved results which agrees with the possibility of an enhanced effect proposed by previous studies (39, 40). In addition, although little was reported about the processes of development of the mHealth messages, there is potential to increase community participation and overall interest in vaccination among mothers and care-givers during the development process especially if it involves development and pilot testing phases (41–43). The small sample sizes of some of the studies and the inclusion criteria of ownership and use of mobile phones by many of them may have introduced a sampling bias in these studies and would have to be considered when adopting these mHealth methods on a population scale (53, 55, 66).

Furthermore, the quality of the studies in this review ranged from RCT to quasi-experimental, and intervention studies as well as cross-sectional/descriptive studies. The quality of these studies and the rigor in their methodologies had some impact on the conclusions by the authors. Although the conclusion of a positive effect of mHealth interventions on vaccination outcomes were made by both RCT and non-RCT studies, the RCT studies had more assertive conclusions.

Overall, SMS reminders for vaccination appointments were found to increase vaccination uptake and reduce delays in receiving vaccinations with a direct association in all but two of the studies. In certain instances, monetary incentives proved beneficial as mothers who received an incentive reported mostly positive experiences with receiving SMS reminders about vaccination appointments (64, 65).However, in contrast, a study from Pakistan found no significant difference in the response rates to SMS messages about vaccinations, even when a financial inducement was introduced (59).

We observed that education and behavioral change because of mHealth interventions was the most represented domain, to which 21 of the reviewed papers related (56–73). Mobile technology makes it easier to contact individuals and offers a useful tool to deliver education and improve health-seeking behavior or health-related lifestyle decisions. However, there was no qualitative study in the review to explore other factors that may have been responsible for any observed change.

Although the review was not conducted to compare external factors related to success of vaccination programs, our findings indicated some level of success irrespective of economic status and the study setting. The impact of this study is that it could inform policymakers and practitioners in LMIC that adopting the use of mHealth may be a low hanging fruit in improving vaccination coverage in their countries.

### 4.1 Strengths and limitations

One of the major strengths of this study was our search strategy, which had a wide timeframe, included 5 databases, and involved a variety of countries, populations, and study designs. However, our search did not include all databases and was restricted to studies conducted between 1st January 2000 and 31st October 2024. Although this search period was adopted because of reports of an increase in mobile phone use based on previous studies (9–13), we concede that it may have resulted in exclusion of some important studies. Moreover, our selection criteria included only studies which were published in the English language. This might have resulted in absence of potentially relevant studies conducted in other languages such as French and Spanish which are widely spoken in LMIC. During the review, a total of 254 studies were excluded which is an indication for greater refining of search strategies in future reviews. Furthermore, given the regular changes to vaccination data, our findings may not be generalizable a few years down the line.

## 5 Conclusion

This scoping review identified and described several studies used to demonstrate a relationship between mHealth and vaccination coverage outcomes. The review shows that mHealth technologies in combination with other interventions have been used to increase vaccination uptake in LMIC. The review also identified that the results of most studies broadly suggest an improved uptake of vaccinations with mHealth especially mobile phone–based interventions. However, there is a need for further research to adequately quantify the impact of these interventions and determine the most effective strategies to increase vaccination outcomes.

## Data Availability

The original contributions presented in the study are included in the article/Supplementary material, further inquiries can be directed to the corresponding author/s.
