# Supplementary material for: Mobile health interventions on vaccination coverage among children under 5 years of age in Low and Middle-Income countries; a scoping review
Source: Front Public Health. 2025 Jan 28;13:1392709. doi: 10.3389/fpubh.2025.1392709 (PMC11810739; doi:10.3389/fpubh.2025.1392709)
Supplement: Supplementary file 1 [file Table_1.DOCX]

**Supplementary Table 1: Outline of search strategy for each database**

**Primary Search Strategy for PubMed**

Date of search – 22^nd^ November 2024

Specific research question: "effects of mHealth interventions on vaccination outcome in low- and middle-income countries"

Database selection: "PubMed"

Keywords:

#1 Immunization [MeSH Terms] OR immunize OR vaccine

#2 child OR teenager OR infant* OR caregiver OR under-five OR mother OR pregnant

#3 cellphone OR “mobile phone” OR “text message” OR “short message service” OR “social media” OR eHealth OR reminder OR “telemedicine” [MeSH Terms]

#4 randomized controlled trial OR controlled clinical trial OR observational studies NOT animals

#5 Africa OR Asia OR South-East Asia OR Sub-Sahara* Africa OR Far East AND Middle East OR Latin America OR Hispanic OR Caribbean Islands OR Low- and Middle-Income Countries OR LMICs

#6 #1 AND #2 AND #3 AND #4 AND #5

#7 #6 with Filters and restrictions: English language publications only. Year – 1^st^ January 2000 to 31^st^ October 2024.

**Primary Search Strategy for Web of Science**

Date of search – 22^nd^ November 2024

Specific research question: "effects of mHealth interventions on vaccination outcome in low- and middle-income countries"

Database selection: "Web of Science"

Keywords:

#1 TOPIC: ("immunization” OR “immunize” OR “immunize”)

#2 TOPIC: ("child” OR “infant” OR “mother” OR “caregiver” OR “under-five” OR “pregnant”)

#3 TOPIC: (“cellphone” OR “mobile phone” OR “SMS” OR text message” OR “short message service”)

#4 TOPIC: (“randomized controlled trial” OR “controlled clinical trial”)

#5 TOPIC: (“Africa” OR “Asia” OR “Far East” AND “Middle East” OR “Latin America” Caribbean Islands OR “Low- and Middle-Income Countries” OR “LMIC”)

#6 #1 AND #2 AND #3 AND #4 AND #5

#7 #6 refined by: LANGUAGES: (ENGLISH) AND PUBLICATION YEARS: (2000 OR 2001 OR 2002 OR 2003 OR 2004 OR 2005 OR 2006 OR 2007 OR 2008 OR 2009 OR 2010 OR 2011 OR 2012 OR 2013 OR 2014 Or 2015 OR 2016 OR 2017 OR 2018 OR 2019 OR 2020 OR 2021 OR 2022 OR 2023 OR 2024)

**Primary Search Strategy for SciDirect**

Date of search – 22^nd^ November 2024

Specific research question: "effects of mHealth interventions on vaccination outcome in low- and middle-income countries"

Database selection: "SciDirect"

Keywords: #1 Immunization OR immunis* OR vaccine

#2 child OR infant* OR caregiver OR under-five OR mother OR pregnant

#3 cellphone OR “mobile phone” OR “text message” OR “short message service” OR “text reminder” OR SMS OR eHealth

#4 randomized controlled trial OR controlled clinical trial

#5 Africa OR Asia OR South-East Asia OR Sub-Sahara* Africa OR Far East AND Middle East OR Latin America OR Hispanic OR Caribbean Islands OR Low- and Middle-Income Countries OR LMIC

#6 #1 AND #2 AND #3 AND #4 AND #5

Filters and restrictions: English language publications only. Year – 1^st^ January 2000 to 31^st^ October 2024.

**Primary Search Strategy for Embase**

Date of search – 22^nd^ November 2024

Specific research question: "effects of mHealth interventions on vaccination outcome in low- and middle-income countries"

Database selection: "Embase"

#1 Immunization OR immunize OR vaccin*

#2 child OR infant* OR caregiver OR pregnant OR mother

#3 cellphone OR “mobile phone” OR “text message” OR SMS

#4 randomized controlled trial OR clinical trial

#5 Africa OR Asia OR South-East Asia OR Sub-Sahara* Africa OR Far East AND Middle East OR Latin America OR Hispanic OR Caribbean Islands OR Low- and Middle-Income Countries OR LMIC

#6 #1 AND #2 AND #3 AND #4 AND #5

Filters and restrictions: English language publications only. Year – 1^st^ January 2000 to 31^st^ October 2024.

**Primary Search Strategy for CINAHL**

MH = Exact Subject Heading

Date of search – 22^nd^ November 2024

Specific research question: "effects of mHealth interventions on vaccination outcome in low- and middle-income countries"

Database selection: "CINAHL"

#1 (MH "immunization")

#2 child OR infant* OR caregiver OR under-five

#3 cellphone OR “mobile phone” OR “text message” OR “short message service” OR “text reminder”

#4 #1 OR #2 Or #3

#5 Sub-Sahara* Africa OR Far East AND Middle East OR Latin America OR Hispanic OR Caribbean Islands OR Low- and Middle-Income Countries OR LMIC

#6 (MH "Clinical trial")

#7 #4 OR #5 OR #6

#8 #7 Peer Reviewed Published Date: 1^st^ January 200-31^st^ October 2024. Narrowed by Language: -English

**Primary Search Strategy for Cochrane**

Date of search – 22^nd^ November 2024

Specific research question: "effects of mHealth interventions on vaccination outcomes in low- and middle-income countries"

Database selection: "Cochrane"

Keywords:

#1 Immunization OR immunise OR vaccin*

#2 infant* OR caregiver OR under-five OR mother OR pregnant

#3 cellphone OR “mobile phone” OR “text message” OR “short message service” OR “text reminder” OR SMS

#4 randomized controlled trial OR observational studies NOT animals

#5 Africa OR Asia OR South-East Asia OR Sub-Sahara* Africa OR Far East AND Middle East OR Caribbean Islands OR Low- and Middle-Income Countries OR LMIC

#6 #1 AND #2 AND #3 AND #4 AND #5

Filters and restrictions: English language publications only. Year – 1^st^ January 2000 to 31^st^ October 2024.
